# Distinguishing cytotoxicity-associated and direct immunomodulatory effects of enniatins and beauvericin in human immune and intestinal cells

**DOI:** 10.1007/s12550-026-00660-2

**Published:** 2026-07-13

**Authors:** Ibrahim Elesh, Dino Grgic, Lada Ivanova, Vanessa Partsch, Christiane Kruse Faeste, Sonja Hager, Francesco Crudo, Doris Marko

**Affiliations:** 1 Department of Food Chemistry and Toxicology, Faculty of Chemistry, University of Vienna, Währinger Str. 38-40, Vienna, 1090 Austria; 2 Doctoral School in Chemistry, Faculty of Chemistry, University of Vienna, Währinger Str. 38-40, Vienna, 1090 Austria; 3 Norwegian Veterinary Institute, P.O. Box 64, Ås, 1431 Norway

**Keywords:** Enniatins, Beauvericin, Immunomodulation, Cytotoxicity, Monocytes, Intestinal cell lines

## Abstract

Enniatins (ENNs) and beauvericin (BEA) are emerging *Fusarium* mycotoxins frequently detected in cereals and processed foods, raising concerns about their toxic potential. These cyclic hexadepsipeptides exert cytotoxic effects by disrupting ionic homeostasis and accumulating in lipid membranes. Although ENNs and BEA modulate pathways such as NF-κB and MAPK, their direct immunomodulatory effects remain poorly characterized. This study investigates their impact on inflammation, cytokine regulation, and metabolic activity in human immune (THP-1 Lucia™ monocytes) and intestinal (HCEC-1CT, Caco-2) cell models, including effects of phase I metabolites generated by S9 mix preincubation. In THP-1 Lucia™ monocytes, all tested ENNs (2.5-5 µM) and BEA (5 µM) suppressed lipopolysaccharide-induced NF-κB activity only at cytotoxic concentrations, suggesting that immunosuppressive effects are secondary to cell damage. Pre-treatment with rat liver S9 mix mitigated cytotoxicity and attenuated NF-κB suppression. In intestinal cells, ENNs and BEA reduced *TNF-α* transcription in non-tumorigenic HCEC-1CT but not in tumorigenic Caco-2 cells, indicating cell-type-specific responses. These findings emphasize the context-dependent immunotoxicity of ENNs and BEA and underscore the need to distinguish between direct immunomodulation and secondary effects of reduced cell viability. While broader immune disruption appears limited to toxic concentrations, *TNF-α* suppression in non-tumorigenic intestinal cells may pose a health concern.

**Supplementary Information:**

The online version contains supplementary material available at 10.1007/s12550-026-00660-2.

## Introduction

Mycotoxins are secondary metabolites that are synthesized by various fungal species. Production of specific mycotoxins by fungi is influenced by environmental conditions, including temperature, moisture levels, oxygen availability, and nutrient composition. These factors determine the profile and concentration of toxins synthesized and can lead to subsequent contamination of food and feed commodities. Dietary exposure to mycotoxins has been demonstrated to induce a broad spectrum of both acute and chronic health effects (Creppy 2002; Long et al. 2025). Consequently, their presence in foodstuffs should be minimized as much as feasible. Therefore, the European Commission has established maximum levels for certain mycotoxins (e.g., aflatoxins, deoxynivalenol, zearalenone, and ochratoxin A) in specific food products (European Commission 2018). 

Enniatins (ENNs) and beauvericin (BEA), however, are not yet regulated in the European Union because of insufficient data on their toxic potentials. These secondary metabolites are classified as ‘emerging’ mycotoxins, as further investigations are required to assess possible adverse health effects connected to dietary exposure. Consequently, new data on ENNs and BEA toxicity have to be provided so that regulatory bodies can decide on potential future regulations with regard to food and feed. According to the European Food Safety Authority (EFSA), there is a necessity for comprehensive risk assessment of these compounds, due to their presence in grains and products thereof (e.g. bakery goods, bread and pasta), which act as common sources of chronic dietary exposure (EFSA Panel 2014; Knutsen et al. 2024). Reported concentrations generally range from low µg/kg levels up to several hundred µg/kg, with highly contaminated samples reaching the mg/kg range. ENN B was detected in cereal-based products at concentrations up to 1100 µg/kg (Juan et al. 2013), while wheat samples showed levels up to 815 µg/kg (Stanciu et al. 2017). In Belgian wheat, ENN B and ENN B1 were consistently detected, with maxima of 2168 and 776.7 µg/kg, respectively (Bertero, et al., 2020). BEA has been reported in corn-derived products at concentrations up to 1006.56 µg/kg, with average levels around 65 µg/kg (Han et al. 2019). 

**Fig. 1:**
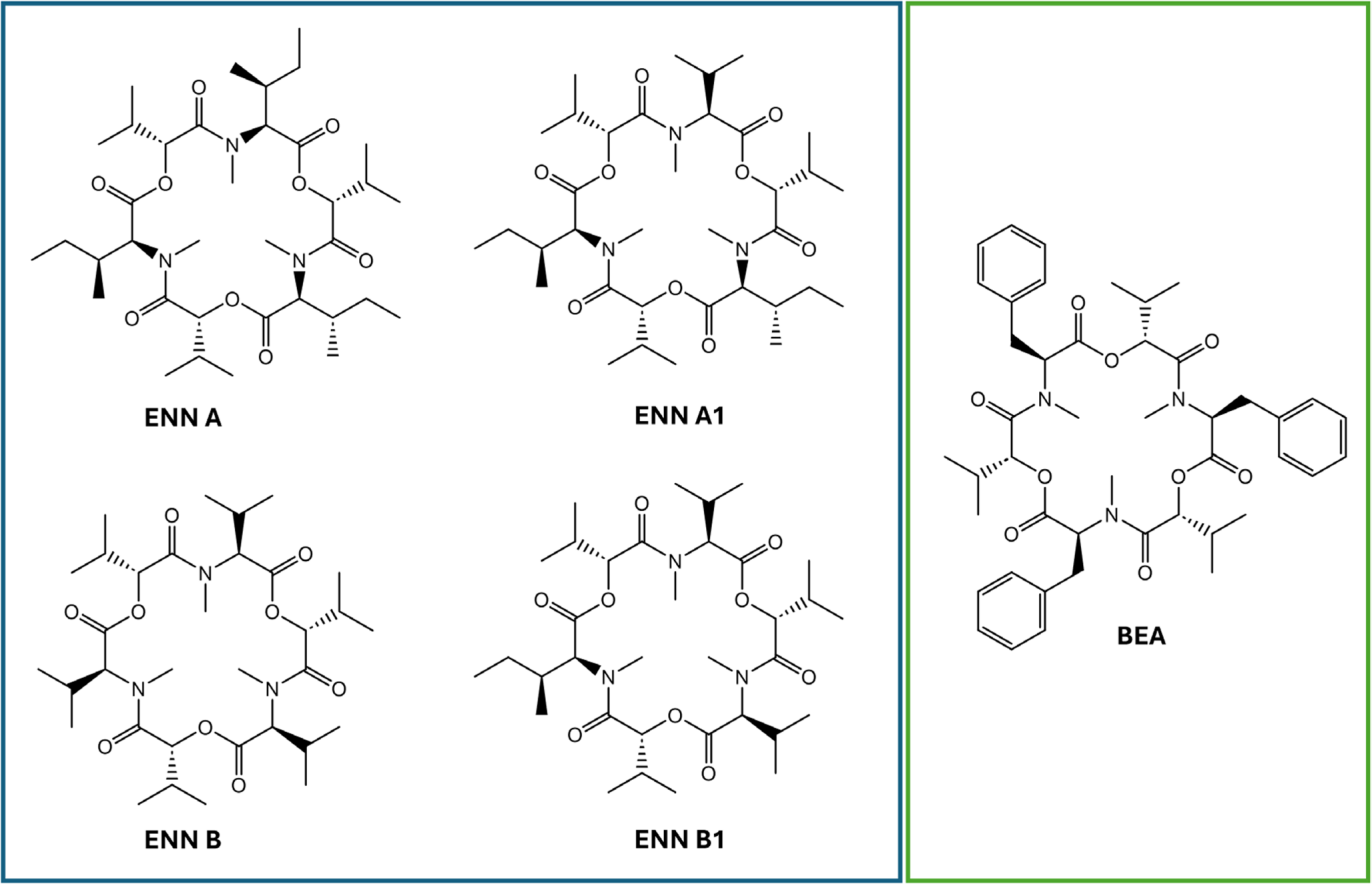
Chemical structures of selected enniatins (ENNs) and beauvericin (BEA)

Structurally, ENNs and BEA belong to the class of cyclic hexadepsipeptides, comprising three α-D-hydroxyisovaleric acid residues alternately connected to three L-configured N-methylated amino acid residues. The nature of the amino acid side chains confers the structural and functional specificity of each compound (Strongman et al. 1988) (Fig. 1).

Their lipophilic structure enables them to incorporate into membranes, where they form transmembrane cation-selective channels. This mechanism is considered the primary mode of action underlying the observed toxicological effects (Prosperini et al. 2017; Štellerová et al. 2023). By increasing membrane permeability towards cations, these compounds thus result in the collapse of the cation gradients (Kouri et al. 2003; Tonshin et al. 2010; Štellerová et al. 2023). The disturbance of ion homeostasis, such as of monovalent (Na^+^, K^+^) and divalent cations (Ca^2+^), triggers the activation of caspases, induces disruption of the mitochondrial membrane potential and enhances reactive oxygen species (ROS) production, which subsequently induces apoptosis (Tedjiotsop Feudjio et al. 2010). 

Recently, the immunotoxic properties of ENNs and BEA have been investigated in various in vitro and in vivo models using different immune cell types. It has been demonstrated that these emerging mycotoxins have the capacity to modulate immune cells by inducing the release of proinflammatory cytokines and impairing macrophage endocytosis (Behr et al. 2025). In dendritic cells, the immunomodulatory effects observed were contradictory, with either a promotion of anti-inflammatory signals and reduction of maturation, or the stimulation of proinflammatory cytokines. The actual outcome seems to be dependent on the exposure dose and duration (Behr et al. 2025). Furthermore, studies indicate that these mycotoxins have the capacity to modify cytokine levels and T cell profiles in concentration- and sex-dependent manner, which can occasionally result in immunosuppression and compromise immune function (Tedjiotsop Feudjio et al. 2010; Tonshin et al. 2010; Gammelsrud et al. 2012; Behr et al. 2025). 

Growing awareness about the significance of immunotoxicity as a toxicological endpoint of foodborne contaminants will eventually lead to a more profound comprehension of concentration-dependent immunomodulation processes. Resulting adverse health effects could include an increased vulnerability towards infections due to immunosuppression. Thus, addressing these knowledge gaps is critical to perform appropriate risk assessments for the ENNs and BEA and can help establish evidence-based regulatory frameworks that protect human and animal health against invisible threats. Considering the high prevalence of these mycotoxins in food- and feedstuffs, the investigation of their potential systemic toxicity is highly relevant and a prerequisite of subsequent regulatory measures (Tedjiotsop Feudjio et al. 2010). However, comprehensive studies evaluating the immunotoxicological potential of ENNs and BEA remain scarce (Bertero et al. 2020). Immunotoxicological effects caused after oral uptake might manifest either directly in the gastrointestinal tract or systemically if the compounds are absorbed into the blood stream. Therefore, the aim of this study was to investigate the dose-dependent cytotoxic and immunomodulatory effects induced by ENNs and BEA, on human immune and intestinal (non- and tumorigenic) cell models to better characterize their toxicological impacts. 

## Materials and methods

### Mycotoxin stock solution

ENNs (ENN A, ENN A1, ENN B, and ENN B1) and BEA, each with a purity of ≥ 98%, were synthesized and provided by Prof. Roderich Süßmuth (Technical University (TU) Berlin, Germany). Stock solutions were prepared by dissolving the compounds in sterile dimethyl sulfoxide (DMSO; Sigma–Aldrich, St. Louis, USA) at a concentration of 10 mM and aliquots were distributed into sterile amber glass vials and stored immediately at −80 °C in accordance with the standard operating procedures (SOPs) agreed within the WP5.1.1.a Hazard assessment group in the Partnership for Assessment of Risk from Chemicals (PARC) to ensure optimal stability and structural integrity. For use in experiments, aliquots were thawed and diluted to adequate levels. The final DMSO concentration under all experimental conditions was not exceeding 0.1% for HCEC-1CT and Caco-2 cells, and 0.25% for THP-1 monocytes. 

The concentration range of 0.01–5 µM was selected as an exploratory range for hazard identification. This selection was based on the limited availability of quantitative toxicokinetic data for ENNs and BEA in human target tissues, particularly immune tissues; previous in vitro studies reporting intestinal transport, bioavailability and cellular effects in the low micromolar range, and preliminary cytotoxicity testing performed in the present experimental models. Available literature indicates that ENNs and BEA are systemically distributed and can accumulate in several tissues, including serum, liver, adipose tissue, brain, kidney and colon (Rodríguez-Carrasco et al. 2016). However, quantitative concentration data for human immune tissues are currently lacking. Consequently, the applied concentrations were not intended to reflect a defined immune tissue burden but rather to cover a biologically relevant range from non-cytotoxic to near-threshold effect levels for mechanistic hazard characterization. The same nominal concentration range was initially applied across the different cell models to enable comparison of relative cellular sensitivity. In contrast, subsequent intestinal gene and protein expression analysis were restricted to non-cytotoxic concentrations (0.1 and 1 µM) in order to avoid potential confounding effects caused by general cytotoxicity. 

### Cell culture

THP-1 Lucia™ NF-κB reporter monocytes, undifferentiated HCEC-1CT and differentiated Caco-2 clone C2BBe1 cells were selected to evaluate the immunomodulatory effects of ENNs and BEA in human immune and intestinal (tumorigenic and non-tumorigenic) cell culture models. THP-1 Lucia™ NF-κB reporter monocytes (InvivoGen, San Diego, CA, USA) were cultured in RPMI 1640 medium containing 10% fetal calf serum (FCS), 1% penicillin/streptomycin (P/S; 100 U/mL and 100 µg/mL, respectively), 25 mM HEPES (Thermo Fisher Scientific, Vienna, AUT) and 100 µg/mL Normocin™ (Invivogen, San Diego, USA) at 37 °C in a humidified atmosphere with 5% CO₂. Zeocin^®^ (100 µg/mL) (Invivogen, San Diego, USA) was added every second passage (up to 25 passages) to maintain the functional integrity of the NF-κB fluorescence reporter system. The non-tumorigenic human colonic epithelial cell line HCEC-1CT, generously provided by Prof. Jerry W. Shay (UT Southwestern Medical Center, Dallas, TX, USA), was cultured in Dulbecco’s Modified Eagle Medium (DMEM, high glucose; Thermo Fisher Scientific, Vienna, AUT) supplemented with 2% HyClone™ Cosmic Calf™ serum, 2% 10× Medium 199, 20 mM HEPES buffer, 50 µg/mL gentamicin, 1 µg/mL hydrocortisone, 10 µL/mL insulin-transferrin-selenium and 20 ng/mL recombinant human epidermal growth factor (EGF) (Thermo Fisher Scientific, Vienna, AUT). The cells were maintained in a humidified atmosphere with 5% CO₂ at 37 °C and passaged every 3 to 4 days at a confluence of 70%. The Caco-2 clone C2BBe1 (ATCC CRL-2102), derived from human colorectal adenocarcinoma and capable of differentiating spontaneously into a polarized monolayer with an apical brush border when cultured to confluence, was obtained from the American Type Culture Collection (ATCC; Manassas, VA, USA). Cells were maintained under humidified conditions (37 °C, 5% CO₂) in DMEM supplemented with 10% FCS, 1% P/S, 1% sodium pyruvate, and 0.01 mg/mL transferrin (Thermo Fisher Scientific, Vienna, AUT). Sub-culturing was performed twice weekly upon reaching approximately 80% confluency. THP-1 monocytes were used as a human innate immune cell model to investigate NF-κB-dependent inflammatory signaling. In addition, HCEC-1CT and differentiated Caco-2 cells were applied as complementary intestinal epithelial models representing non-tumorigenic and enterocyte-like phenotypes at the primary site of dietary mycotoxin exposure. 

### CellTiter®-Blue assay

The CellTiter^®^-Blue (CTB) assay (Promega Corp., Fitchburg, USA) was carried out to assess effects on the metabolic activity and determine the general cytotoxicity of ENNs and BEA in the three cell models (THP1-Lucia™ monocytes, HCEC-1CT and Caco-2 (C2BBe1)) to ensure that the effects observed in subsequent immunomodulatory assays were not only artefacts due to cytotoxicity. HCEC-1CT (5000 cells/well) and Caco-2 (24650 cells/well) were seeded for 2 and 7 days, respectively, in a 96-well plate. Cells were incubated with 100 µL ENNs or BEA at concentrations ranging from 0.01 to 5 µM, a solvent control (0.1% DMSO) and Triton-X as positive control to induce cell death for 2 h, followed by immune stimulation with recombinant human interleukin-1 beta (IL-1β, 25 ng/mL, InvivoGen, San Diego, CA) diluted in endotoxin-free water for additional 18 h. In THP-1 Lucia™ monocytes, 100 µL ENNs or BEA (0.01–5 µM), solvent control (0.25% DMSO) or the cell death control (0.1% Triton-X) were added to each well. Subsequently, 100 µL THP1-Lucia™ cell suspension (0.1 × 10⁶ cells/well) were dispensed into the wells and incubated for 2 h. Lipopolysaccharide (LPS; 10 ng/mL) was added to all wells to stimulate immune responses, followed by additional 18 h incubation. At the end of the incubation period, the culture medium was replaced by 10% CTB reagent solution on the plates containing HCEC-1CT and Caco-2 cells. For THP-1 Lucia™ cells, 20 µL CTB were added directly. All cells were then incubated for 2 h at 37 °C in a humidified atmosphere containing 5% CO₂. Following the incubation, THP1-Lucia™ monocytes were centrifuged at 140 × *g* for 2 min before 100 µL (for THP1-Lucia™) or 80 µL (for HCEC-1CT and Caco-2) culture supernatant was transferred into black 96-well plates for fluorescence measurement. Fluorescence was quantified using a Gen5 Microplate Reader (BioTek, Vienna, Austria) at an excitation wavelength of 530 nm and an emission wavelength of 560 nm. 

### NF-κB reporter gene assay

The NF-κB reporter gene assay was performed in 96-well plates. First, 100 µL ENNs or BEA (0.01–5 µM), solvent control (0.25% DMSO) and a control for immunosuppression (1 µM dexamethasone (DEXA)) were added to each well. Subsequently, 100 µL THP1-Lucia™ cells (0.1 × 10⁶ cells/well) were dispensed into the wells and incubated for 2 h. LPS (10 ng/mL) was added to all wells to induce NF-κB activation, followed by additional 18 h incubation. At the end of the incubation period, the plates were centrifuged at 140 × *g* for 2 min, and 20 µL of the supernatant were transferred into a white 96-well plate. Next, 50 µL QUANTI-Luc™ reagent (InvivoGen, USA) were added and luminescence was measured immediately using the Cytation 3 Cell Imaging Multi-Mode Reader (BioTek, Vienna, Austria). 

### Preincubation with S9 mix 

The NF-κB reporter gene assay and CTB assays were performed with and without preincubation with S9 mix. The S9 mix, a rat liver enzyme preparation competent for xenobiotic metabolism, was used to evaluate whether biotransformation may modify the extent of the immunotoxic effects of ENNs and BEA on THP1-Lucia™. Sprague-Dawley rat liver S9 (product number: S2067) was obtained from Sigma-Aldrich (Merck, St. Louis, MO). The S9 mix was prepared by adding KH₂PO₄ (100 mM), MgCl₂ (8 mM) and NADPH (4 mM) as cofactors to the S9 preparation (25 mg/mL). The S9 fraction was diluted with the respective supplements to obtain an S9 working solution containing 2.5 mg/mL S9 protein, of which 200 µL were mixed with 800 µL of the toxin solution, resulting in a final incubation volume of 1 mL, a final S9 working solution content of 20% v/v and a final S9 protein concentration of 0.5 mg/mL. ENNs and BEA (5 µM) were incubated with and without S9 mix for 2 h at 37 °C. The samples were centrifuged at 9000 × *g* for 5 min, and the supernatants were subsequently used in the different assays.

### Mass spectrometry analysis

#### Sample preparation

ENN B, B1 and BEA were incubated with S9 mix as described above and then diluted at a ratio of 1:1 (v/v) with ice-cold ACN. Thereafter, the samples were centrifuged at 20,000 × *g* and 4 °C for 10 min. The resulting supernatants (80 µL) were filtered using Thomson Single StEP nano filters (Thomson, Carlsbad, CA, USA) prior to UPLC–HRMS/MS analysis. In addition, a sample combined of aliquots from incubation replicates was prepared for each mycotoxin with the aim of increasing the chance to detect low-abundance metabolites. Of this “pooled S9 sample”, 300 µL were extracted three consecutive times with 500 µL ethyl acetate (EA) by vortexing for 10 s per extraction. The combined EA fractions were evaporated at 40 °C under a gentle stream of nitrogen and the dried residues were reconstituted in 50 µL 50% (v/v) ACN in water and analyzed by HRMS/MS. To facilitate metabolite identification, defined metabolite extracts generated in human liver microsomes (HLM) on two independent days were included in the analysis as references (Ivanova et al., 2024). 

#### UPLC-HRMS/MS method

Samples from the S9 experiments were analyzed by ultra-performance liquid chromatography high-resolution tandem mass spectrometry (UPLC–HRMS/MS) for the detection of potential metabolites of ENN B, ENN B1 and BEA. HRMS analysis was performed utilising an Orbitrap Fusion Tribrid mass spectrometer equipped with a heated electrospray ion source (HESI-II) coupled to an Ultimate 3000 UHPLC system (both by Thermo Fisher Scientific, San Jose, CA, USA). The HESI-II interface was operated in positive ion mode at 300 °C; other instrument parameters were adjusted as following: spray voltage 3.8 kV, heated transfer capillary temperature 280 °C, sheath gas flow rate 35 L/min, auxiliary gas flow rate 10 L/min, S-lens RF level 60. The full scan experiments ranged from 600 to 1000 *m/z*, with a selected resolution of 120,000. For the MS^2^ method, precursors were directly identified in the MS^1^ scan employing a targeted mass difference of 4.9554 Da for sodiated and ammoniated molecular ions. Fragmentation experiments performed by collision-induced dissociation (CID) and higher-energy C-trap dissociation (HCD) were based on sodiated molecular ions, which yielded the most informative MS^2^ spectra, which were recorded at 30,000 resolution with an isolation window of 1.4 *m/z*. The HCD collision energy was normalized and set to 50, the AGC target was 100% and the maximum injected time 80 ms. The CID collision energy was fixed at 35%. 

Chromatographic separation was performed on a Phenomenex Kinetex^®^ F5 column (2.1 × 100 mm, 1.7 μm; Phenomenex, Torrance, CA, USA) maintained at 30 °C. Parent compounds and biotransformation products were eluted during a 30-min analytical run. The mobile phases consisted of water (A) and methanol (B), each containing 2 mM ammonium formate and 2 mM formic acid. Analytes of interest were separated by gradient elution starting with 5% B for 1.7 min, followed by a linear increase to 40% B for 6.7 min, elution at 40% B for 2 min, a further increase to 70% B at 20.7 min, and subsequently to 100% B at 24.7 min. After maintaining 100% B for 2 min, the column was re-equilibrated for 3 min at 5% B prior to the next injection. The flow rate was 0.4 mL/min and a 1 µL sample was injected. 

#### Data processing

Data processing Raw files, including the solvent control, mycotoxin reference standards, mixtures of ENN B-, ENN B1- or BEA-related metabolites formed in HLM and pooled S9 samples were processed in Compound Discoverer 3.3 SP3 (Thermo Fisher Scientific) for the tentative annotation by matching retention time, mass and fragmentation data to the entries in a previously established in-house MzVault 2.3 SP1 library generated from HLM incubations (Ivanova et al., 2024). Molecular networking was applied to identify metabolites related to the parent compounds. The in-house library was used to annotate known metabolites with a mass tolerance of 5 ppm and a retention time (RT) tolerance of 0.15 min. Metabolites were assigned different levels of annotation confidence. Low-confidence annotations were based solely on the presence of sodiated ([M + Na]^+^ and/or or ammonium adducts [M+NH_4_]^+^) without sufficient HRMS² data. High-confidence annotations were supported by informative MS² spectra of [M + Na]^+^ ions contained in the in-house library. 

### Quantitative real-time PCR (qRT-PCR)

Undifferentiated HCEC-1CT cells (60.000 cells per well in 12-well plates) and Caco-2 cells (155.000 cells per well in 24-well plates) were seeded and cultured for 48 h and 7 days, respectively, before treatment with ENNs or BEA. To investigate the immunomodulatory impact of the mycotoxins on intestinal inflammation, cells were pre-incubated with non-cytotoxic concentrations of ENNs and BEA (0.1 and 1 µM) for 2 h followed by co-incubation with 25 ng/mL IL-1β for an additional 3 h. The supernatant was collected and stored at −80 °C for further analysis as described in Sect. “Enzyme-linked immunosorbent assay (ELISA)”. Total RNA was extracted following the RNeasy Mini Kit protocol from Qiagen (Hilden, Germany). RNA quantity and purity were assessed spectrophotometrically. RNA concentrations ranged between 102 and 268 ng/µL, and purity ratios were between 1.98 and 2.09 for both A260/A280 and A260/A230. Reverse transcription of 1 µg RNA to cDNA was carried out in accordance with the QuantiTect^®^ Reverse Transcription manual. Thereafter, qRT-PCR was performed using the StepOne Plus PCR System (Applied Biosystems, Thermo Fisher Scientific). For each reaction, 20 ng cDNA were amplified using the MiScript SYBR Green PCR Kit (Qiagen). Gene expression was determined for the following targets using QuantiTect^®^ primer assays (Qiagen): GAPDH (Hs_GAPDH_1_SG, QT00079247), β-Actin (Hs_ACTB_1_SG, QT00095431), TNF-α (Hs_TNF_1_SG, QT00029162), IL-6 (Hs_IL6_1_SG, QT00083720), IL-8 (Hs_CXCL8_1_SG, QT00000322) and IL-10 (Hs_IL_1_SG, QT00041685). After activating the polymerase at 95 °C for 15 min, the samples were processed in 40 cycles consisting of cDNA denaturation at 94 °C for 15 s, annealing of the primers at 55 °C for 30 s and extension through new DNA synthesis at 70 °C for 30 s. The measured results of all samples were normalized to the mean expression of the endogenous control genes GAPDH and β-actin, and relative quantification was performed using the 2^⁻ΔΔCt^ method, yielding fold-change values relative to the solvent control (Livak et Schmittgen 2001).

### Enzyme-linked immunosorbent assay (ELISA)

ELISA was used to assess the concentration of TNF-α in the supernatant of the mycotoxin-exposed intestinal cells described in “Quantitative real-time PCR (qRT-PCR)”. The ELISA (TNF alpha Human Uncoated ELISA Kit, product number: 88–7346-88, Invitrogen, Thermo Fisher Scientific) was performed according to the manufacturer’s instructions. 

### Statistical analysis

Effects of the different mycotoxins in the NF-κB reporter gene assay and CTB were investigated in technical triplicates (repeated measurements with the same cell passage) and at least four independent biological replicates (*n* ≥ 4) sourced from different cell passages. Biological replicate values were tested for outliers using the Nalimov test prior to statistical analysis. The test was applied within each treatment group at α = 0.05. Values were excluded only when the calculated Nalimov q-value exceeded the corresponding critical value for the respective number of replicates at the 95% confidence level. Outlier exclusion was not based on visual inspection alone, and no more than one biological replicate was removed per treatment group. qRT-PCR and ELISA experiments were performed in technical duplicates and at least three independent biological replicates (*n* ≥ 3). The mean values of the biological replicates were tested for normality using the Shapiro-Wilk test. The mean values of the biological replicates were tested for normality using the Shapiro-Wilk test. Statistical analyses and plotting of data were performed with the software Origin Pro^®^ 2025 (OriginLab, Northampton, MA, USA). Significant differences were identified by one-way analysis of variance (ANOVA) followed by Bonferroni post hoc correction for multiple comparisons. Significance levels of 5%, 1% and 0.1% were indicated respectively as * = *p* < 0.05, ** = *p* < 0.01 and *** = *p* < 0.001, or # = *p* < 0.05, ## = *p* < 0.01 and ### = *p* < 0.001. 

## Results

### THP-1 Lucia™

#### Cell viability measured as metabolic activity 

**Fig. 2:**
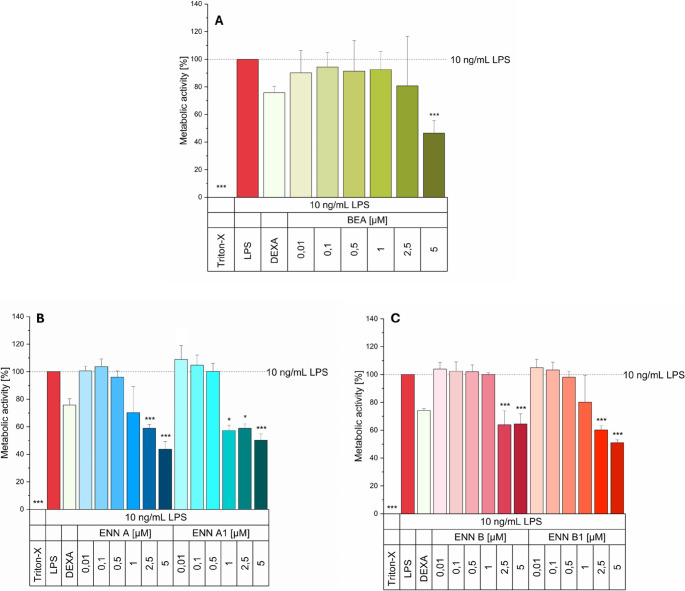
Effects of **A**) beauvericin (BEA), and **B**) and **C**) enniatins (ENNs) on the viability [%] of THP1-Lucia™ monocytes as measured in the CellTiter® Blue (CTB) assay after 20 h incubation with rising mycotoxin concentrations. Values were normalized to the control (10 ng/mL LPS), which was set to 100%. Triton-X (0.1%) was used as a positive control for cell death, whereas 1 µM dexamethasone (DEXA) served as an immunosuppressive control. Results are depicted as mean + standard deviation of n ≥ 4 biological replicates, each performed in triplicate after the exclusion of outliers. Significant differences are indicated by*(p < 0.05) and *** (p < 0.001)

CTB metabolic activity assays were conducted in LPS-stimulated THP-1 Lucia™ NF-κB cells (Fig. 2) to differentiate between effects caused by the general toxicity of ENNs and BEA and specific immunomodulation in the subsequently performed functional assays. The CTB experiment showed that BEA exhibited only minor reduction in cell viability at concentrations up to 2.5 µM and significantly reduced metabolic activity only to 46% at the highest tested concentration (5 µM), suggesting a limited cytotoxic potential in THP-1 Lucia™ cells (Fig. 2 A). In contrast, ENNs (ENN A, ENN A1, ENN B and ENN B1) reduced the metabolic activity in a concentration-dependent manner, with a significant reduction in metabolic activity observed at ≥ 2.5 µM (ENN A (*p* < 0.0001), A1 (*p* = 0.027), B (*p* = 0.00042) and B1 (*p* < 0.0001)) (Fig. 2B and 2 C). Among the tested ENNs, ENN A1 exerted the most pronounced cytotoxic effect, reducing metabolic activity to 57%, 58%, and 50% at concentrations of 1, 2.5, and 5 µM, respectively. ENN A significantly decreased metabolic activity to 58% at 2.5 µM (*p* < 0.0001), and 43% at 5 µM (*p* < 0.0001), showing clear concentration-effect dependency. ENN B reached a plateau of at maximum 63% metabolic activity reduction at 2.5 µM (*p* = 0.00042), whereas ENN B1 had a similar effect profile as ENN A, exhibiting concentration-dependent effects with 80% at 1 µM, 60% at 2.5 µM (*p* < 0.0001) and 55% at 5 µM (*p* < 0.0001).

#### NF-_k_B reporter gene assay

**Fig. 3:**
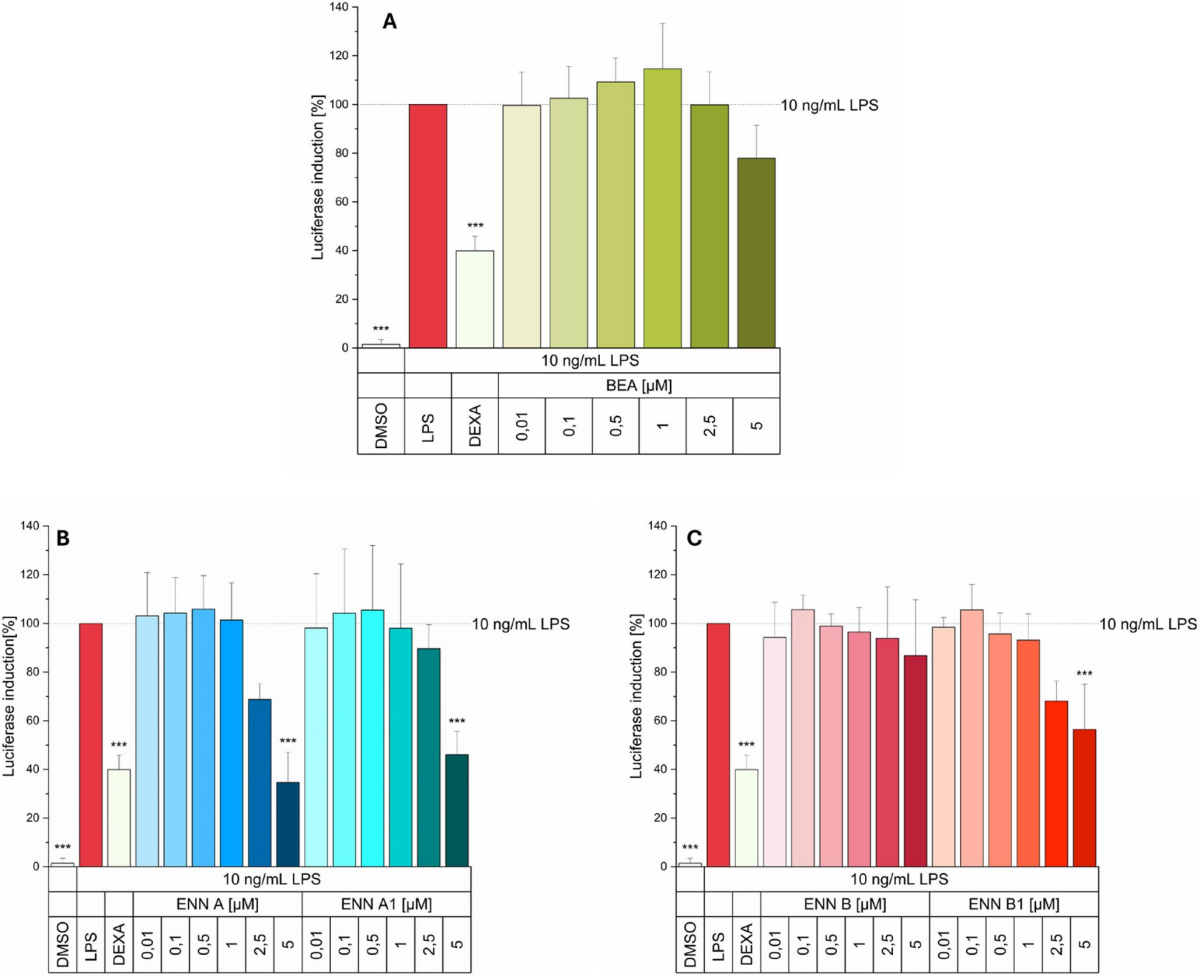
Effects of **A**) beauvericin (BEA), and **B**) and **C**) enniatins (ENNs) on the NF-κB signal transduction pathway in THP1-Lucia™ cells after 20 h incubation with rising mycotoxin concentrations. Values were normalized to the control (10 ng/mL LPS) as 100%. The solvent control DMSO (0.25%) was not treated with LPS. Triton-X (0.1%) was used as a positive control for cell death, whereas 1 µM dexamethasone (DEXA) served as an immunosuppressive control. Results are depicted as mean + standard deviation of n ≥ 4 biological replicates, each performed in triplicate after the exclusion of outliers. Significant differences are indicated by *** (p < 0.001)

The immunomodulatory potential of ENNs and BEA was evaluated by examining their attenuation on the NF-κB pathway in LPS-stimulated THP-1 Lucia™ monocytes. The cells contain a stable NF-κB-inducible luciferase reporter gene, which produces a quantifiable fluorescence signal after activation of different transmembrane pattern-recognition receptors that activate NF-κB signal transduction. BEA had minimal impact on NF-κB activity across the tested concentration range, with only a minor reduction to 80% observed at 5 µM. However, this effect was not statistically significant (Fig. 3A). In general, ENNs were found to reduce NF-κB activity in a concentration-dependent manner, with significant reduction observed at ≥ 5 µM (Figs. 3B and 3 C). ENN A exhibited the strongest effect, reducing NF-κB signal transduction to 68% and 34% at 2.5 and 5 µM (*p* < 0.0001), respectively. ENN A1 had a significant effect only at 5 µM (*p* = 0.00024, 46% signal reduction) (Fig. 3B). In comparison, ENN B1 reduced NF-κB activity to 68% and 56% at 2.5 and 5 µM (*p* = 0.00064), respectively, whereas ENN B did not significantly affect NF-κB activity at any of the tested concentrations (Fig. 3C). Data for ENNs and BEA in non–LPS-stimulated THP-1 Lucia™ monocytes are shown in Supplementary Figs. 1 and 2.

#### Assessment of S9 preincubation for metabolic activation in in vitro systems

**Fig. 4:**
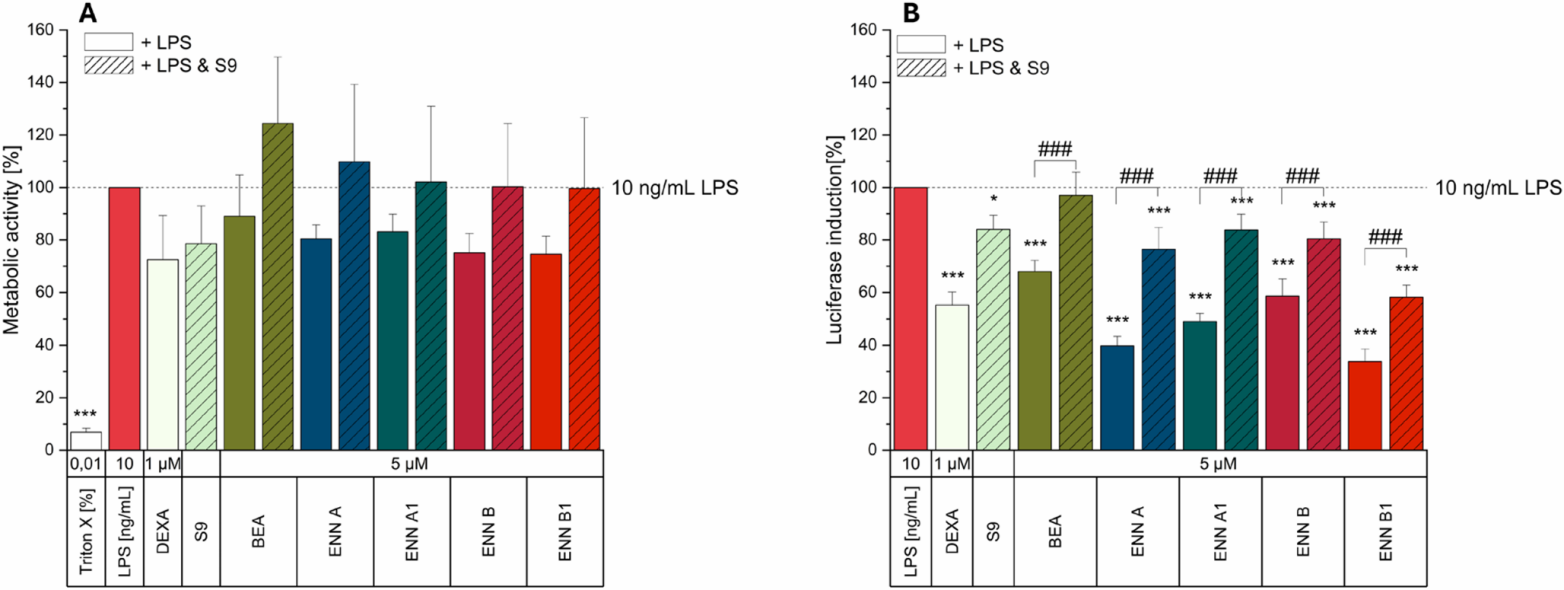
Impact of liver S9 mix preincubation of enniatins (ENNs) and beauvericin (BEA) (5 µM) on **A**) cell viability and **B**) NF-κB signal transduction pathways in THP1-Lucia™ cells after 20 h incubation. Values were normalized to the control (10 ng/mL LPS) as 100%. Triton-X (0.1%) was used as a positive control for cell death, whereas 1 µM dexamethasone (DEXA) served as an immunosuppressive control. Results are depicted as mean + standard deviation of n ≥ 3 biological replicates, each performed in technical triplicates. Significant differences between mycotoxins and the LPS control are indicated by*(p < 0.05) and ***(p < 0.001), whereas significant differences between S9-treated (striped bars) and -untreated (clear bars) mycotoxins are indicated with ###(p < 0.001)

To further investigate potential immunomodulatory and cytotoxic effects after metabolic transformation, the impact of ENNs and BEA on both NF-κB activation and cytotoxicity was examined following preincubation with a rat S9 mix, allowing comparison between the parent compounds and their formed phase I metabolites. Accordingly, the cytotoxic potential of S9-incubated ENNs and BEA was decreased in LPS-stimulated THP-1 Lucia™ cells as compared to the untreated mycotoxins at 5 µM in the CTB metabolic activity assay in LPS -stimulated THP-1 Lucia™ cells (Fig. 4**A**). The decrease was notable but not statistically significant, and the least relevant for BEA because the effect of the non-metabolized compound was already small. S9 mix alone was slightly cytotoxic, reducing the metabolic activity of the monocytes to about 80% A modulating effect by S9-preincubation of the mycotoxins was also observable in the NF-κB signal transduction assay (Fig. 4B). While the parent compounds showed at 5 µM the expected substantial decrease (*p* < 0.001) in luciferase induction, this effect was significantly less pronounced after S9-pretreatment of ENNs and BEA, indicating a reduced immunomodulatory effect of the S9-treated mycotoxins. The S9 mix alone reduced NF-κB-associated pathways by about 20%.

#### Characterization of ENN B, ENN B1 and BEA metabolites with S9 mix

Phase I metabolites of ENN B, ENN B1 and BEA formed with rat liver S9 mix were tentatively characterized by UPLC-HRMS/MS analysis and comparison to existing in-house libraries that had been established from incubations with human liver microsomes. The metabolite concentrations in the S9 incubations performed in the present study were generally low and replicate samples had to be pooled and concentrated to allow metabolite annotation. All identified metabolites had shorter retention times as compared to their respective parent compounds, confirming that phase I biotransformation products generally become more polar through the introduction of hydrophilic functional groups in typical reactions such as hydroxylation, demethylation and oxidation. Based on accurate mass measurements in HRMS, MS² fragmentation patterns and retention times, several metabolites could be annotated with varying levels of confidence. For ENN B, five metabolites were identified with high confidence (Table 1). Demethylated M32 was the most abundant metabolite in the chromatogram of the pooled S9 sample, followed by M26, M27 and M25 (Supplementary Fig. 3). Two notable peaks at retention time (RT) 16.4 min and 17.5 min in the rat liver S9 chromatogram had no comparable counterparts in HLM and could thus not be annotated.

**Table 1:** Tentatively identified phase I metabolites of ENN B produced by incubation with rat liver S9

ID^1^	RT (min)	Observed mass [M + Na^+^] (m/z)	Transformation	Molecular formula	Confidence
M9	12.1	664.3773	-(CH_2_)+(O)	C_32_H_55_N_3_O_10_	low
M11	12.5	664.3768	-(CH_2_)+(O)	C_32_H_55_N_3_O_10_	low
M13	12.7	664.3771	-(CH_2_)+(O)	C_32_H_55_N_3_O_10_	low
M14	13.0	664.3773	-(CH_2_)+(O)	C_32_H_55_N_3_O_10_	low
M16	13.3	664.3774	-(CH_2_)+(O)	C_32_H_55_N_3_O_10_	low
M18	13.4	664.3772	-(CH_2_)+(O)	C_32_H_55_N_3_O_10_	low
M19	13.7	676.3777	− (2 H)+(O)	C_33_H_55_N_3_O_10_	low
M21	13.9	664.3771	-(CH_2_)+(O)	C_32_H_55_N_3_O_10_	low
M22	13.9	692.3727	− (2 H)+(2O)	C_33_H_55_N_3_O_11_	low
M23	14.0	678.3923	+(O)	C_33_H_57_N_3_O_10_	low
M24	14.2	678.3921	+(O)	C_33_H_57_N_3_O_10_	*high*
M25	14.2	687.4159 [M+NH_4_]^+^	− (2 H)+(2O)	C_33_H_55_N_3_O_11_	low
M26	14.4	664.3773	-(C H_2_)+(O)	C_32_H_55_N_3_O_10_	low
M27	14.5	678.3931	+(O)	C_33_H_57_N_3_O_10_	*high*
M28	14.8	678.3925	+(O)	C_33_H_57_N_3_O_10_	*high*
M29	15.1	678.3929	+(O)	C_33_H_57_N_3_O_10_	*high*
M30	15.2	678.3925	+(O)	C_33_H_57_N_3_O_10_	low
M31	15.6	634.3667	-(C_2_H_4_)	C_31_H_53_N_3_O_9_	low
M32	16.8	648.3817	-(CH_2_)	C_32_H_55_N_3_O_9_	*high*
ENN B	17.8	662.3973	parent	C_33_H_57_N_3_O_9_	*high*

^1^ENN B metabolites were annotated based on their respective [M + Na]^+^ adducts; however, the confidence in unambiguous identification was low for 74% of the metabolites due to the lack of suitable HRMS² data

ENN B1 was metabolized by rat liver S9 to a number of phase I metabolites that could be annotated by comparison to an in-house library based on HLM data (Table 2). Four metabolites were identified with high confidence. M19 and M20 were the most abundant metabolites in the chromatogram of the pooled S9 sample, followed by M23 (Supplementary Fig. 4). All three were generated by the addition of oxygen to ENN B1 in the rat liver S9.

**Table 2:** Tentatively identified phase I metabolites of ENN B1 produced by incubation with rat liver S9

ID^1^	RT (min)	Observed mass [M + Na^+^] (m/z)	Transformation	Molecular formula	Confidence
M1	11.0	708.4034	+(2O)	C_34_H_59_N_3_O_11_	low
M2	11.2	708.4037	+(2O)	C_34_H_59_N_3_O_11_	low
M3	11.9	708.4042	+(2O)	C_34_H_59_N_3_O_11_	low
M4	12.2	708.4043	+(2O)	C_34_H_59_N_3_O_11_	low
M5	12.4	706.3882	− (2 H)+(2O)	C_34_H_57_N_3_O_11_	low
M7	12.6	706.3887	− (2 H)+(2O)	C_34_H_57_N_3_O_11_	low
M8	12.8	701.4326 [M+NH_4_]^+^	− (2 H)+(2O)	C_34_H_57_N_3_O_11_	low
M9	13.1	678.3938	-(CH_2_)+(O)	C_33_H_57_N_3_O_10_	*high*
M10	13.2	706.3916	− (2 H)+(2O)	C_34_H_57_N_3_O_11_	low
M13	14.0	706.3881	− (2 H)+(2O)	C_34_H_57_N_3_O_11_	low
M14	14.0	692.4087	+(O)	C_34_H_59_N_3_O_10_	low
M15	14.3	706.3882	− (2 H)+(2O)	C_34_H_57_N_3_O_11_	low
M17	14.6	706.3880	− (2 H)+(2O)	C_34_H_57_N_3_O_11_	low
M16	14.7	692.4092	+(O)	C_34_H_59_N_3_O_10_	low
M19	15.1	692.4087	+(O)	C_34_H_59_N_3_O_10_	*high*
M20	15.2	692.4083	+(O)	C_34_H_59_N_3_O_10_	*high*
M21	15.7	690.3929	− (2 H) +(O)	C_34_H_57_N_3_O_10_	low
M22	16.2	690.3932	− (2 H) +(O)	C_34_H_57_N_3_O_10_	low
M23	17.7	662.3992	-(CH_2_)	C_33_H_57_N_3_O_9_	*high*
ENN B1	18.6	676.4135	parent	C_34_H_59_N_3_O_9_	*high*

^1^ENN B1 metabolites were annotated based on their respective [M + Na]^+^ adducts; however, the confidence in unambiguous identification was low for 79% of the metabolites due to the lack of suitable HRMS² data

Similarly, BEA incubation with rat liver S9 produced several phase I metabolites, which could be characterized by comparison to human metabolites generated in HLM (Table 3). Oxidized M11 showed the highest peak in the chromatogram of the pooled S9 sample, but M9 and M13 (both oxidized) as well as M14 (demethylated) were also notably present (Supplementary Fig. 5).

**Table 3:** Tentatively identified phase I metabolites of BEA produced by incubation with rat liver S9

ID^1^	RT (min)	Observed mass [M + Na^+^] (m/z)	Transformation	Molecular formula	Confidence
M1	14.4	835.4485 [M+NH_4_]^+^	+(2 H)+(2O)	C_45_H_59_N_3_O_11_	low
M2	15.0	838.3886	+(2O)	C_45_H_57_N_3_O_11_	low
M3	15.6	838.3892	+(2O)	C_45_H_57_N_3_O_11_	low
M4	15.7	835.0000 [M+NH_4_]^+^	+(2 H)+(2O)	C_45_H_59_N_3_O_11_	low
M5	16.0	838.3887	+(2O)	C_45_H_57_N_3_O_11_	low
M6	16.1	838.3887	+(2O)	C_45_H_57_N_3_O_11_	low
M7	16.8	822.3940	+(O)	C_45_H_57_N_3_O_10_	low
M8	17.1	822.3936	+(O)	C_45_H_57_N_3_O_10_	low
M9	17.5	822.3932	+(O)	C_45_H_57_N_3_O_10_	*high*
M10	17.8	822.2937	+(O)	C_45_H_57_N_3_O_10_	low
M11	18.0	822.3928	+(O)	C_45_H_57_N_3_O_10_	*high*
M12	18.4	822.3941	+(O)	C_45_H_57_N_3_O_10_	low
M13	18.8	822.3934	+(O)	C_45_H_57_N_3_O_10_	low
M14	19.4	792.3829	-(CH_2_)	C_44_H_55_N_3_O_9_	*high*
BEA	20.0	806.3976	parent	C_45_H_57_N_3_O_9_	*high*

^1^BEA metabolites were annotated based on their respective [M + Na]^+^ adducts; however, the confidence in unambiguous identification was low for 79% of the metabolites due to the lack of suitable HRMS² data

### Intestinal epithelial cells (HCEC-1CT, Caco-2)

#### Cell viability (CTB)

**Fig. 5:**
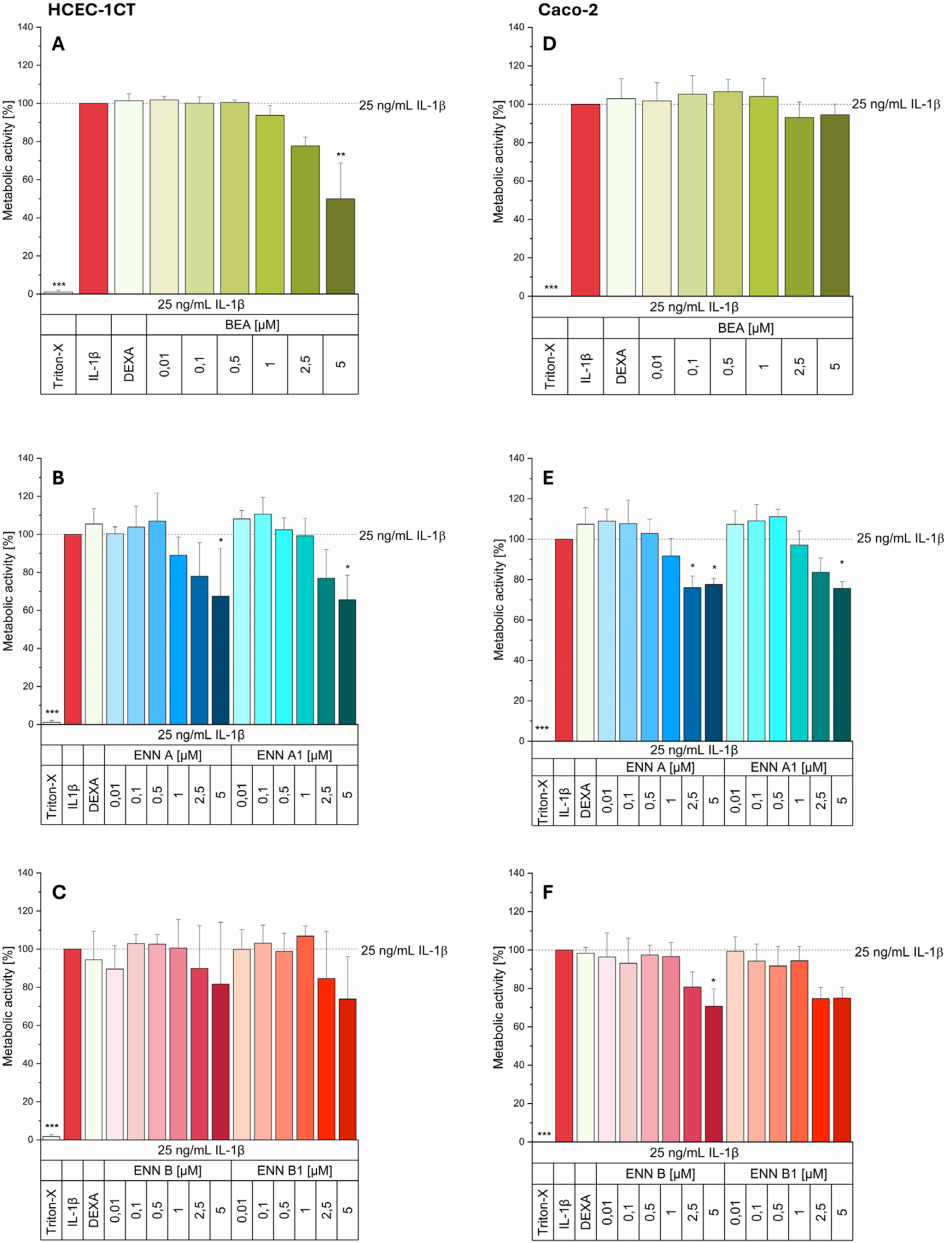
Effects of **A)** and **D**) beauvericin (BEA) and **B**),**C**),**E**), and **F**) enniatins (ENNs) on the viability [%] of HCEC-1CT and Caco-2 cells as measured with the CellTiter Blue (CTB) assay after 20 h incubation with rising mycotoxin concentrations. Values were normalized to the control (25 ng/mL IL-1β) as 100%. Triton-X (0.1%) was used as a positive control for cell death, whereas 1 µM dexamethasone (DEXA) served as an immunosuppressive control. Results are depicted as mean + standard deviation of n ≥ 4 biological replicates, each performed in triplicate after the exclusion of outliers. Significant differences are indicated by*(p < 0.05) and **(p < 0.01)

Cell viability was evaluated in both intestinal cell lines included in this study, the undifferentiated, non-tumorigenic HCEC-1CT cells (Figs. 5A, 5B, 5 C) and differentiated Caco-2 cells (Figs. 5D, 5E, 5F), by measuring metabolic activity under IL-1β stimulation. In HCEC-1CT cells, BEA caused significant cytotoxic effects starting at 5 µM (*p* = 0.0025), whereas no significant cytotoxicity was observed in Caco-2 cells across all tested concentrations (Figs. 5A, 5D). ENN A and ENN A1 reduced metabolic activity in HCEC-1CT significantly only at 5 µM (to 67% and 65%, respectively; Fig. 5B). In Caco-2 cells, ENN A significantly reduced metabolic activity already at 2.5 µM (*p* = 0.024), whereas ENN A1 showed a significant effect only at 5 µM (*p* = 0.024), with both compounds reducing metabolic activity to 71% and 70%, respectively (Fig. 5E). ENN B and ENN B1 were less potent in HCEC-1CT cells and did not significantly affect metabolic activity at any concentration tested, although some effect was visible at higher concentrations (Figs. 5 C, 5 F). In Caco-2 cells, only ENN B induced a significant reduction in metabolic activity at 5 µM (*p* = 0.042), reaching a maximum decrease to 70%, whereas ENN B1 did not cause a statistically significant effect under the tested conditions (Fig. 5F).

#### Quantitative real-time PCR (qRT-PCR)

**Fig. 6:**
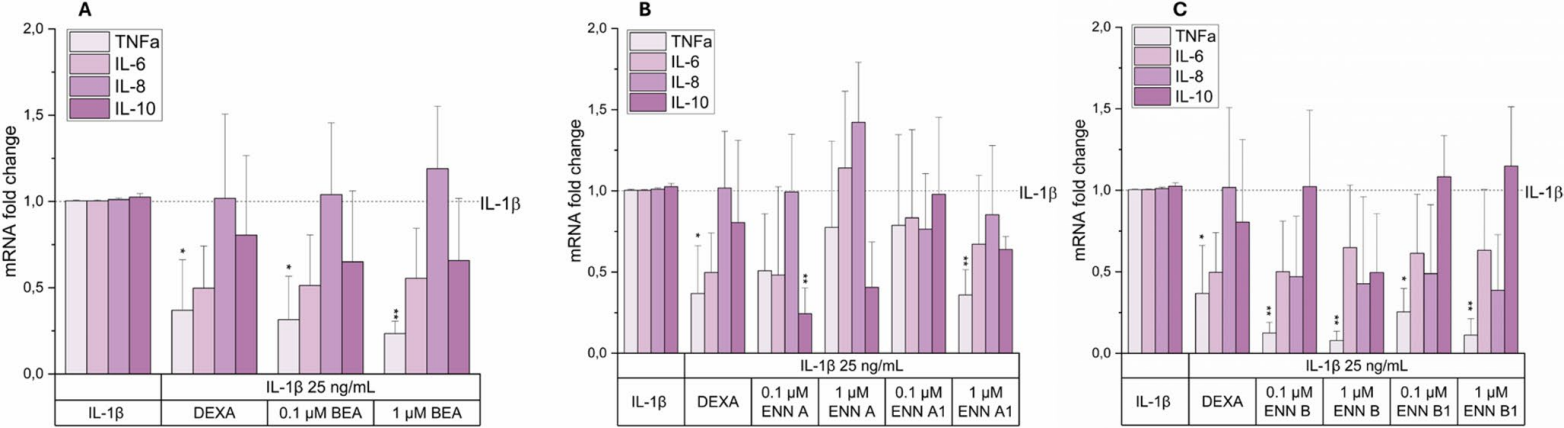
Changes in cytokine mRNA expression in HCEC-1CT cells measured by qRT-PCR after incubation for 2 h with **A**) beauvericin (BEA), and **B**) and **C**) enniatins (ENNs) at 0.1 and 1 µM, followed by co-incubation with 25 ng/mL IL-1β for additional 3 h. Columns represent the fold change of *TNF-α*,*IL-6*,*IL-8* and *IL-10* gene expression, showing the means + standard deviation of at least three independent experiments. Dexamethasone (DEXA) was included as positive control. Significant differences to the IL-1β control are indicated by *(p < 0.05) and **(p < 0.01)

**Fig. 7:**
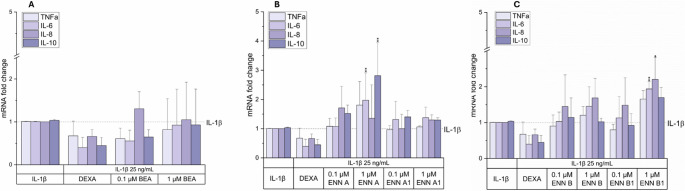
Changes in cytokine mRNA expression in Caco-2 cells measured by qRT-PCR after incubation for 2 h with **A**) beauvericin (BEA), and **B**) and **C**) enniatins (ENNs) at 0.1 and 1 µM, followed by co-incubation with 25 ng/mL IL-1β for an additional 3 h. Columns represent the fold change of *TNF-α*,*IL-6*,*IL-8* and *IL-10* gene transcription, showing the means + standard deviation of at least three independent experiments. Dexamethasone (DEXA) was included as positive control. Significant differences to the IL-1β control are indicated by *(p < 0.05) and **(p < 0.01)

Potential immunomodulatory effects of ENNs and BEA were examined in more detail in the two intestinal cell lines by determining changes in the expression of typical cytokine genes after exposure to the mycotoxins and activation by IL-1β (Figs. 6 and 7). In undifferentiated HCEC-1CT cells, ENNs and BEA demonstrated selective immunomodulatory effects on the cytokine gene expression at non-cytotoxic concentrations (Fig. 6). BEA significantly suppressed *TNF-α* mRNA expression to 0.31-fold and 0.23-fold at 0.1 µM (*p* = 0.044) and 1 µM (*p* = 0.0017), respectively, as compared to IL-1β stimulated controls (Fig. 6A). ENN A reduced TNF-α transcription to 0.5-fold at 0.1 µM. At 1 µM, the result was inconclusive to relatively high variance between the experimental replicates. ENN A1 had practically no effect at 0.1 µM but caused a significant 0.35-fold reduction at 1 µM (0.0073) (Fig. 6B). ENN B exhibited the comparably strongest effect, reducing *TNF-α* mRNA levels to 0.12-fold and 0.10-fold at 0.1 µM (*p* = 0.0035) and 1 µM (*p* = 0.0020), respectively. ENN B1 similarly suppressed TNF-α expression to 0.25-fold at 0.1 µM (*p* = 0.017) and 0.11-fold at 1 µM (*p* = 0.003) (Fig. 6C). In contrast, none of the ENNs and BEA had a significant impact on the mRNA levels of the other tested cytokines (*IL-6*, *IL-8* and *IL10*), although an overall reduction of *IL-6* was observable (Fig. 6). *IL-8* expression remained unchanged (BEA, ENN A, ENN A1) or was reduced (ENN B, ENN B1), whereas *IL-10* was reduced (BEA, ENN A, ENN A1, ENN B) or was unchanged (ENN B1).

In differentiated Caco-2 cells, the effects of ENNs and BEA on cytokine mRNA expression were less pronounced as compared to those observed in undifferentiated HCEC-1CT cells. After 5 h of exposure, BEA caused a slight but insignificant reduction of *TNF-α*, *IL-6* and *IL-10* and an increase of *IL-8* expression at 0.1 µM that were not notable at 1 µM (Fig. 7A). ENN A significantly increased the expression of *IL-6* (*p* = 0.0025) (1.9-fold) and *IL-10* (*p* = 0.0053) (2.8-fold) at 1 µM, whereas ENN A1 had no notable effect on the cytokine expression (Fig. 7B). ENN B showed only a notable, but insignificant increase of *IL-8*. ENN B1 was more potent and significantly elevated *IL-6* (*p* = 0–0069) (1.9-fold), and *IL-8* (*p* = 0.025) (2.2-fold) mRNA levels at 1 µM (Fig. 7C). However, considering a 2-fold change in gene expression as the threshold for biologically relevant upregulation, only ENN A significantly upregulated *IL-10* (2.8-fold), and ENN B1 significantly upregulated *IL-8* (2.2-fold) at 1 µM. None of the other ENNs or BEA induced significant changes in the transcription of the cytokines investigated in this cell model. 

#### Enzyme-linked immunosorbent assay (ELISA)

**Fig. 8:**
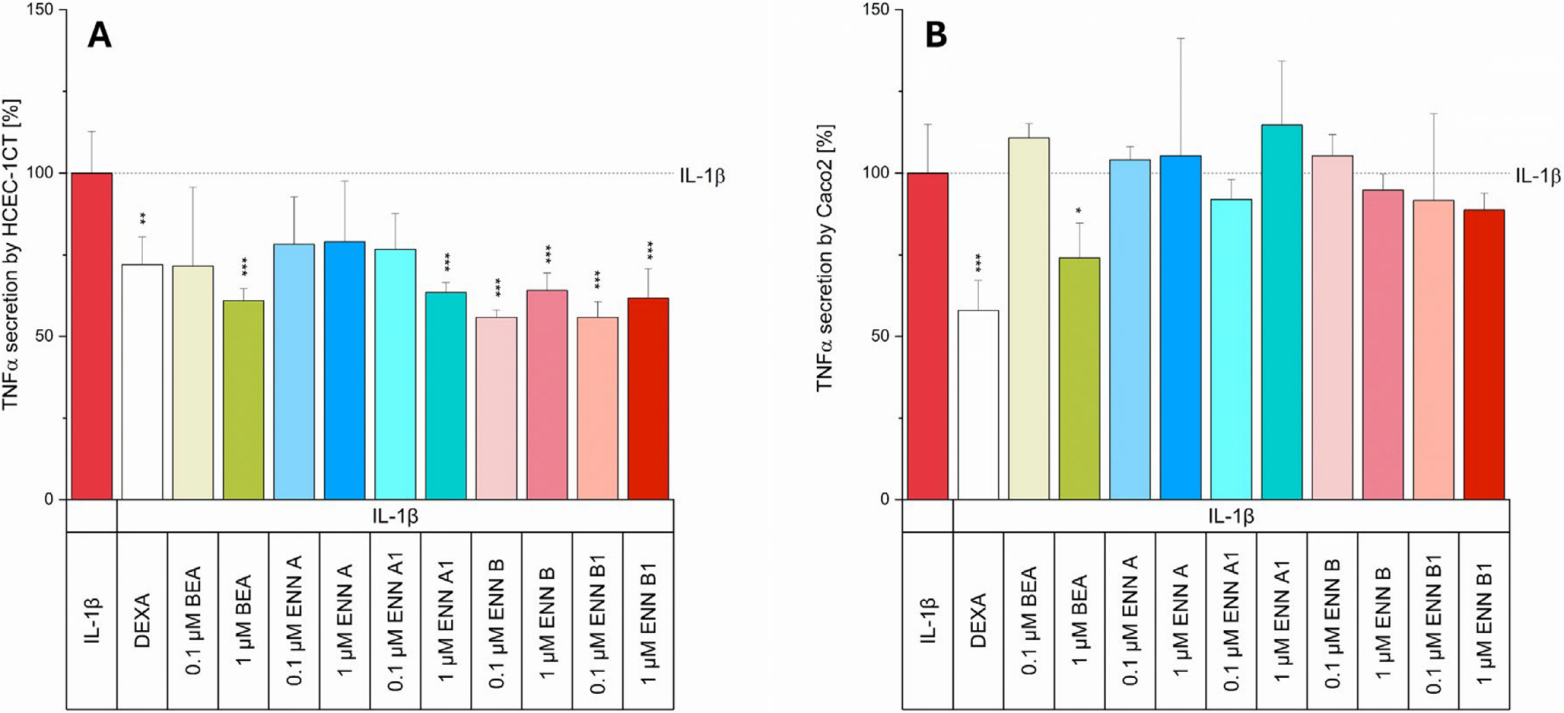
Changes in TNF-α protein expression in **A**) HCEC-1CT and **B**) Caco-2 cells after incubation for 2 h with beauvericin (BEA) and enniatins (ENNs) at 0.1 and 1 µM followed by co-incubation with 25 ng/mL IL-1β for additional 3 h. Dexamethasone (DEXA, 1 µM) served as an immunosuppressive control. Columns represent the levels of TNF-α protein expression, showing the means + standard deviation of at least 3 independent experiments. DEXA was included as positive control. Significant differences to the IL-1β control are indicated by ∗ (p < 0.05), ∗∗ (p < 0.01) and ∗∗∗ (p < 0.001)

ELISA experiments were performed to investigate whether the observed changes in *TNF-α* mRNA levels induced by ENNs and BEA exposure translated into changes in protein concentrations. TNF-α protein was quantified in the supernatant of IL-1β-stimulated HCEC-1CT following exposure to ENNs and BEA (Fig. 8A). All tested compounds significantly reduced (*p* < 0.001) TNF-α protein levels at 0.1 µM and 1 µM as compared to the IL-1β control (set to 100%) with the exception of BEA (0.1 µM), ENN A (both concentrations) and ENN A1 at 0.1 µM. DEXA, used as a control for anti-inflammatory activity, decreased TNF-α protein down to 72%. BEA treatment led to a TNF-α decrease of 72% at 0.1 µM and 61% at 1 µM. ENN A reduced TNF-α protein levels, although with higher inter-sample variability reaching 78% and 79% at 0.1 and 1 µM, respectively. ENN A1 had a more pronounced effect and reduced TNF-α with 77% at 0.1 µM and 63% at 1 µM. However, ENN B and ENN B1 caused even greater reductions at both concentrations, lowering TNF-α levels by 56 to 64%. These findings confirm that the transcriptional downregulation of TNF-α is indeed translated to the protein level and support the immunosuppressive potential of ENNs and BEA in undifferentiated HCEC-1CT cells.

Furthermore, TNF-α concentrations in the supernatant of IL-1β-stimulated Caco-2 cells were measured following exposure to ENNs and BEA (Fig. 8B). DEXA reduced TNF-α protein levels to 55% as compared to the IL-1β-stimulated control. In contrast to the clearly suppressive effects on TNF-α expression in HCEC-1CT cells, the responses in Caco-2 cells were more variable. Only BEA at 1 µM (*p* = 0.033) produced a significant reduction to 71%. At 0.1 µM BEA, a slight increase to 106% was observable, suggesting a concentration-dependent, moderate effect. ENN A and ENN A1 appeared largely ineffective or slightly increased TNF-α protein levels, particularly at 1 µM ENN A1, where expression was raised to 110%. ENN B showed no noteworthy effect at both concentrations, whereas ENN B1 non-significantly reduced TNF-α to 88% and 85% at 0.1 and 1 µM, respectively, with relatively low variability. 

## Discussion

Emerging mycotoxins such as ENNs and BEA are increasingly detected in food and feed, yet their immunotoxicological profiles remain poorly defined. While these mycotoxins are known for their cytotoxic, antimicrobial, and ionophoric activities, their potential to modulate immune responses, particularly at non-cytotoxic concentrations, has not been fully elucidated (Kouri et al. 2003; Tedjiotsop Feudjio et al. 2010; Tonshin et al. 2010; Štellerová et al. 2023). Given that the gastrointestinal tract and the innate immune system are forefront in the defense against dietary xenobiotics, assessing the immunomodulatory effects of these toxins is crucial for understanding possible health risks connected to oral exposure.

In THP-1 monocytes stimulated with LPS, a potent and widely used activator of the NF-κB signaling pathway, exposure to BEA and ENNs significantly suppressed NF-κB-driven luciferase activity in a dose-dependent manner). Notably, all ENNs and BEA were reported to interact with the NF-κB signaling pathway, a central mediator of inflammation, modulating the expression of pro- and anti-inflammatory cytokines (e.g. *TNF-α*,* IL-6*,* IL-8*,* IL-1β* and *IL-10*) (Tedjiotsop Feudjio et al. 2010; Jonsson et al. 2016; Yoo et al. 2017). However, the concurrent reduction in metabolic activity indicates that ENNs and BEA suppressed NF-κB activation only at concentrations above 1 µM, which were associated with reduced metabolic activity. This suggested that the observed suppression is likely a secondary effect of compromised cellular function rather than a direct modulation of the NF-κB signaling pathway. 

Xenobiotic metabolism can reduce the concentrations of active compounds, thereby increasing tolerance to mycotoxin exposure. To explore the influence of biotransformation on the results observed in this study, ENNs and BEA were incubated with rat liver S9 mix before the extract was used in subsequent functional assays. This enabled comparison of effects caused by the parent toxins with those of their phase I metabolites. We observed that the metabolism step significantly diminished the cytotoxic potential of ENNs and BEA, which in turn mitigated NF-κB suppression. 

Phase I metabolites generally result in the formation of hydroxylated, carboxylated and demethylated conjugates, which add polar groups and reduce the hydrophobic properties of the parent compounds (Behr et al. 2025). In the case of ENNs and BEA, the initial lipophilicity of the parent compounds enables their embedding into lipid bilayers, such as cell membranes. This process can result in the formation of channels or pores, through which monovalent ions as well as to some extent Ca^2+^ and Mg^2+^ can pass (Kouri et al. 2003; Štellerová et al. 2023). The subsequent induction of programmed cell death is a consequence of this process (Tedjiotsop Feudjio et al. 2010) Incubation of ENN B, ENN B1 and BEA with S9 mix resulted in the formation of oxidative, more hydrophilic metabolites, which were tentatively identified by UPLC-HRMS/MS and comparison to metabolite libraries generated using human liver microsomes. The experiments indicate that these mycotoxins will undergo biotransformation to less active metabolites in vivo, reducing the levels of toxic parent compounds and thus the risk of adverse health effects. This became evident in the metabolic activity and NF-κB signal transduction assays, where the observed loss in cell viability and initiation of NF-κB signaling caused by the ENNs and BEA was mitigated by the S9 preincubation, indicating that decreased lipophilicity by the introduction of polar groups plays an important role in detoxification. The increased hydrophilicity of the metabolites hinders their integration into cell membranes, thereby preventing the formation of selective ion channels. However, since the metabolites were only tentatively annotated and not isolated or tested individually, conclusions regarding their reduced biological activity remain inferential and are based on the combined observation of metabolite formation and attenuated effects after S9 preincubation. 

Neither ENNs nor BEA exhibited clear immunosuppressive properties in THP-1 monocytes, as the observed effects appeared to be secondary to reduced cell viability. This underscored the importance of distinguishing between true immunomodulatory effects and those arising from cell damage. Overall, the results suggested that ENNs and BEA may pose an immunotoxicological risk primarily at concentrations with reduced cell viability in immune cells, rather than through specific modulation of immune signaling pathways. As THP-1 cells are assumed to represent a model for systemic effects in the blood compartment, the absence of specific immunotoxicity, particularly after metabolic transformation, suggests that the systemic immune risk after exposure is low. 

The possible impact of ENNs and BEA on intestinal cells after oral uptake, especially considering a modulation of immune responses in the digestive tract, was evaluated in experiments using undifferentiated non-tumorigenic HCEC-1CT cells and differentiated Caco-2 cells. Intestinal epithelial cells are the primary site of contact after oral exposure and play a critical role in modulating local and systemic immune functions through cytokine release and maintenance of barrier integrity. Determining the sensitivities of both cell lines to the mycotoxins in the metabolic activity assay showed that the HCEC-1CT cells exhibited greater resilience to ENN-induced cytotoxicity than the differentiated Caco-2 cells, which were significantly more affected at equal concentrations. This finding is consistent with reports describing that ENNs exert potent cytotoxic effects on cancer-derived cell lines at low micromolar concentrations (Olleik et al. 2019; Bertero et al. 2020), while non-tumorigenic cells often withstand higher ENN concentrations (Olleik et al. 2019). In contrast, BEA exerted a distinct cytotoxic effect with a significant reduction in metabolic activity starting from 2.5 µM in HCEC-1CT cells, whereas no cytotoxicity was induced in Caco-2 cells up to 5 µM. This might be attributed to certain ABC transporters, such as ABCG2, which moderately reduced BEA-induced toxicity but had little effect on the potency of ENNs (Dornetshuber et al. 2009).

Analyzing these differences in more depth, the impact of ENNs and BEA on cytokine expression in the two intestinal cell lines was assessed via RT-qPCR analyses targeting *TNF-α*,* IL-6*,* IL-8*, and *IL-10*. At non-cytotoxic concentrations, the mycotoxins exhibited different immunomodulatory effects depending on the cell type and specific cytokine. In HCEC-1CT cells, all ENNs and BEA significantly downregulated *TNF-α* expression following IL-1β stimulation, indicating a suppressive effect on pro-inflammatory signaling, meaning that they have a dampening effect on the immune response. The changes in mRNA expression were substantiated at the protein level as determined by ELISA, which demonstrated a decrease in TNF-α secretion. In line with these findings, studies utilizing a co-culture model with macrophages have repeatedly demonstrated that BEA significantly reduces cytokine levels in epithelial cells, thereby substantiating its immunosuppressive properties (Shandilya et al. 2023; Behr et al. 2025). This immunosuppressive effect was also observed in vivo in colitis-induced mice, where intraperitoneal administration of BEA led to a significant decrease in TNF-α serum levels (Wu et al. 2013). None of the other cytokines investigated in the present study (*IL-6*,* IL-8*, and *IL-10*) were significantly affected in HCEC-1CT cells upon treatment with ENNs and BEA. The cytokine levels stayed unchanged or were slightly reduced, but without reaching significance, except for ENN A suppressing *IL-10.*

In Caco-2 cells, no substantial downregulation of cytokine gene expression or TNF-α protein levels was observed in general. However, BEA caused some reduction of TNF-a, whereas exposure to ENNs resulted in an overall induction of cytokine expression. In particular, ENN A led to a moderate upregulation of *TNF-α*, *IL-6*, and *IL-10*, while ENN B1 induced a moderate increase in *TNF-α*, *IL-6*, and *IL-8* expression. These changes did not exceed a two-fold threshold, except for *IL-10* (ENN A) and *IL-8* (ENN B1), which showed more pronounced upregulation. These findings align with previous reports, in which ENNs and BEA were demonstrated to enhance the secretion of IL-8 in epithelial cells when administered in mixtures (Albonico et al. 2016). Furthermore, in a mature dendritic cell model differentiated from human umbilical cord blood mononuclear cells, ENN B and BEA were capable of enhancing IL-10 secretion during the maturation process (Ficheux et al. 2013). 

The opposing results observed for the various cell types in this study indicate that ENNs and BEA modulate immune signaling tissue-dependently. This resulted in compound-specific immunosuppressive effects in HCEC-1CT cells, while no significant changes were exhibited in Caco-2 cells. The substantial effects observed in undifferentiated intestinal cells and THP-1 monocytes exemplified the capacity of these mycotoxins to disrupt immune responses by altering signal transduction pathways. There is growing evidence that *Fusarium* mycotoxins may have the capacity to alter cytokine profiles and immune cell function (Shandilya et al. 2023). The presence of immune mediators such as IFN-γ, IL-8, and IL-10 in co-cultured bovine epithelial cells and macrophages was significantly decreased by ENN B after LPS stimulation. Furthermore, it has been reported that ENN B reduces the phagocytic activity of bovine leukocytes and impairs their ROS production, which might lead to a compromised innate immune function and increased infection risks (Sandrini et al. 2025).

The observation of a decrease in TNF-α levels in HCEC-1CT induced by ENNs and BEA indicates at least a partial disruption of cytokine homeostasis. TNF-α, a cytokine that plays a pivotal role in cell signaling and chemotaxis, is a critical mediator of immune responses against infections. A reduction in TNF-α signaling has been demonstrated to result in a decrease in host resistance to infections (Crawford et Curtis 2008). This increased susceptibility to bacteria and viruses has already been described for other immunosuppressive mycotoxins in animal models (Antonissen et al. 2014; Sun et al. 2023). The hypothesis that consistent exposure to immunosuppressive mycotoxins could potentiate vulnerability to infectious diseases is predicated on the premise that the immune system is weakened over time. For instance, a recent study on bovine immune cells demonstrated that ENN B exposure reduced the neutrophils’ ability to phagocytose bacteria, which might correlate with an increased risk of infection in those animals (Sandrini et al. 2025). 

The concentration range of 0.01–5 µM was selected for the experimental investigation of mechanistic hazard characterization. Limited availability of quantitative data on ENNs and BEA concentrations in human immune tissues was the basis for this selection, despite the existence of evidence indicating systemic distribution and accumulation in several organs (Rodríguez-Carrasco et al. 2016; Behr et al. 2025). Post-oral exposure, local intestinal concentrations may exceed plasma levels, and efficient transepithelial transport has been demonstrated in Caco-2 models, supporting the use of low micromolar concentrations in vitro (Prosperini et al. 2012). ENNs and BEA are commonly detected in cereal-based foods in the µg/kg range, while highly contaminated samples can reach concentrations of approximately 170–1100 µg/kg for ENN B and up to 1006.56 µg/kg for BEA (Juan et al. 2013; Stanciu et al. 2017; Han et al. 2019). These levels correspond to approximately 0.27–1.72 µmol/kg food for ENN B and 1.28 µmol/kg food for BEA. Thus, the non-cytotoxic concentration of 0.1 µM, and to some extent 1 µM, may be relevant for local intestinal exposure under high-contamination scenarios. In contrast, the cytotoxicity-associated NF-κB suppression observed in THP-1 Lucia™ monocytes at ≥ 1–5 µM is less likely to reflect realistic systemic exposure and should rather be interpreted as an upper-bound hazard scenario. Direct extrapolation remains limited by gastrointestinal dilution, matrix effects, absorption, metabolism, and protein and lipid binding. To avoid potential confounding effects due to cytotoxicity, functional analyses were restricted to non-cytotoxic concentrations (0.1–1 µM), whereas higher concentrations were considered to represent an upper-bound hazard range. However, the in vivo relevance of these findings remains uncertain, particularly in view of the current toxicokinetic data gaps highlighted by EFSA (EFSA Panel, 2014). Accordingly, the present study was designed as a mechanistic hazard identification approach to characterize potential immunomodulatory effects of ENNs and BEA, rather than as a quantitative human risk assessment based on realistic internal exposure concentrations.

A limitation of the present study is that the mechanistic analysis focused on immunotoxicologically relevant functional endpoints, including NF-κB activation and cytokine expression and secretion, whereas upstream stress pathways were not experimentally addressed. Although ROS formation, mitochondrial dysfunction, disturbed ion homeostasis and MAPK-related signaling may contribute to ENN- and BEA-induced cellular effects and inflammatory regulation, detailed investigation of these pathways was outside the scope of the present study. Future studies should therefore address these mechanisms to further clarify the upstream events underlying the observed cytokine modulation. 

In conclusion, this study provides insights into the immunomodulatory effects of ENNs and BEA, revealing distinct effects on cell viability, immunomodulation and cytokine secretion in human (non-) tumorigenic immune and intestinal cell models. Differential cell sensitivities under inflammatory conditions highlight the context-dependent nature of the toxicity of ENNs and BEA. In addition, this study underscores the importance of differentiating direct immunomodulatory effects from those arising secondary to cytotoxicity when evaluating emerging mycotoxins. Our data suggests that direct immunomodulation and cytotoxicity-associated secondary effects may coexist, depending on the cell type, concentration and endpoint investigated. At realistic exposure levels, ENNs and BEA showed limited broad immunomodulation, with TNF-α suppression in intestinal epithelial cells at non-cytotoxic concentrations standing out as the main target. These insights contribute to a more nuanced hazard characterization of ENNs and BEA, suggesting that risk assessments should account for their capacity to selectively impair crucial cytokine signals (such as TNF-α) in the gut, even in the absence of overt cytotoxicity. Moreover, effects and associated risks might be moderated in vivo by biotransformation processes that generate less toxic metabolites. Broader changes of immune responses are likely to occur only at doses that substantially compromise cell viability. 

## Supplementary Information

Below is the link to the electronic supplementary material.

Supplementary Material 1 (DOCX 1.23 MB)

## Data Availability

Data will be made available on request.

## Abbreviations

ABC: ATB-Binding cassette
ABCG2: ATB-Binding cassette sub-family G member 2
ACN: Acetonitrile
ATP: Adenosine triphosphate
BEA: Beauvericin
CTB: CellTiter®-Blue
DEXA: Dexamethasone
DMSO: Dimethyl sulfoxide
EA: Ethyl acetate
EGF: Epidermal growth factor
ELISA: Enzyme-linked immunosorbent assay
ENN A: Enniatin A
ENN A1: Enniatin A1
ENN B: Enniatin B
ENN B1: Enniatin B1
ENNs: Enniatins
EFSA: European Food Safety Authority
HCEC-1CT: Human colonic epithelial cells-1CT
HRMS²: High-resolution tandem mass spectrometry
IL-1β: Interleukin-1 beta
IL-6: Interleukin-6
IL-8: Interleukin-8
IL-10: Interleukin-10
LPS: Lipopolysaccharide
NF-κB: Nuclear factor kappa B
PARC: Partnership for Assessment of Risk from Chemicals
P/S: Penicillin/streptomycin
ROS: Reactive oxygen species
TNF-α: Tumor necrosis factor alpha
UHPLC: Ultra-high-performance liquid chromatography
