# Supplementary material for: Distinguishing cytotoxicity-associated and direct immunomodulatory effects of enniatins and beauvericin in human immune and intestinal cells
Source: Mycotoxin Res. 2026 Jul 13;42(3):50. doi: 10.1007/s12550-026-00660-2 (PMC13364816; doi:10.1007/s12550-026-00660-2)
Supplement: Supplementary file 1 — Supplementary Material 1 (DOCX 1.23 MB) [file 12550_2026_660_MOESM1_ESM.docx]

Supplementary information of

**Distinguishing cytotoxicity-associated and direct immunomodulatory effects of enniatins and beauvericin in human immune and intestinal cells**

Ibrahim Elesh ^1,#^, Dino Grgic^1,#,*^, Lada Ivanova^3^, Vanessa Partsch^1,2^, Christiane Kruse Fæste^3^, Sonja Hager^1^, Francesco Crudo^1^, Doris Marko^1^

^1^Department of Food Chemistry and Toxicology, Faculty of Chemistry, University of Vienna, Währinger Str. 38-40, 1090 Vienna, Austria

^2^Doctoral School in Chemistry, Faculty of Chemistry, University of Vienna, Währinger Str. 38-40, 1090 Vienna, Austria

^3^ Norwegian Veterinary Institute, P.O. Box 64, 1431 Ås, Norway

^#^shared first co-authors

^*^corresponding author: [dino.grgic@univie.ac.at](mailto:dino.grgic@univie.ac.at)

ORCID ID:

Ibrahim Elesh: 0000-0001-6529-241X

Dino Grgic: 0000-0001-8753-7027

Lada Ivanova: 0000-0002-4240-2492

Vanessa Partsch: [0009-0000-4073-7013](https://orcid.org/0009-0000-4073-7013)

Sonja Hager: 0000-0001-8221-1044

Francesco Crudo: 0000-0002-4876-8057

Christiane Kruse Fæste: 0000-0001-7820-2882

Doris Marko: 0000-0001-6568-2944

1. The effects of ENNs and BEA in non–LPS-stimulated THP-1 Lucia™ monocytes

**
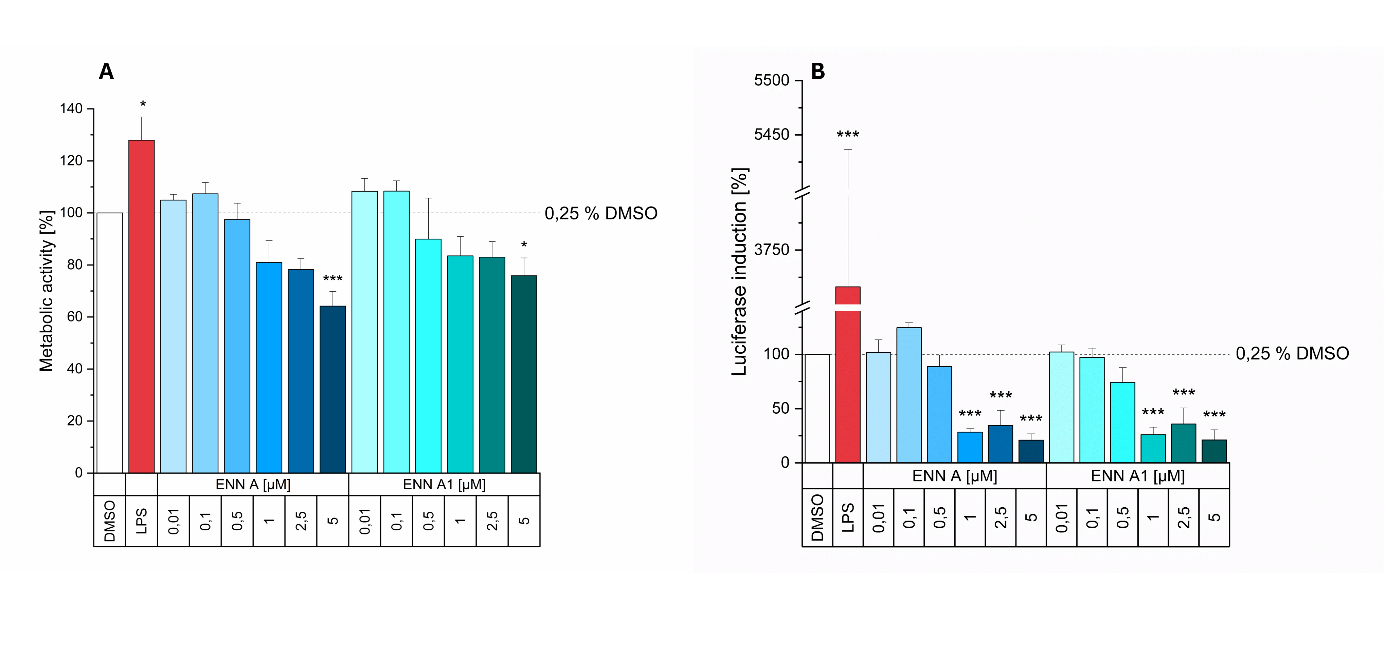
**

**Figure 1**. Impact of enniatin A and A1 (ENN) **A**) cell viability and **B**) NF-κB signal transduction pathways in non–LPS-stimulated THP1-Lucia™ cells after 20 h incubation. Values were normalized to the control (0.25 % DMSO) as 100 %. Results are depicted as mean + standard deviation of n ≥ 3 biological replicates, each performed in technical triplicates. Significant differences between mycotoxins and the control are indicated by *(p < 0.05) and ***(p < 0.001).


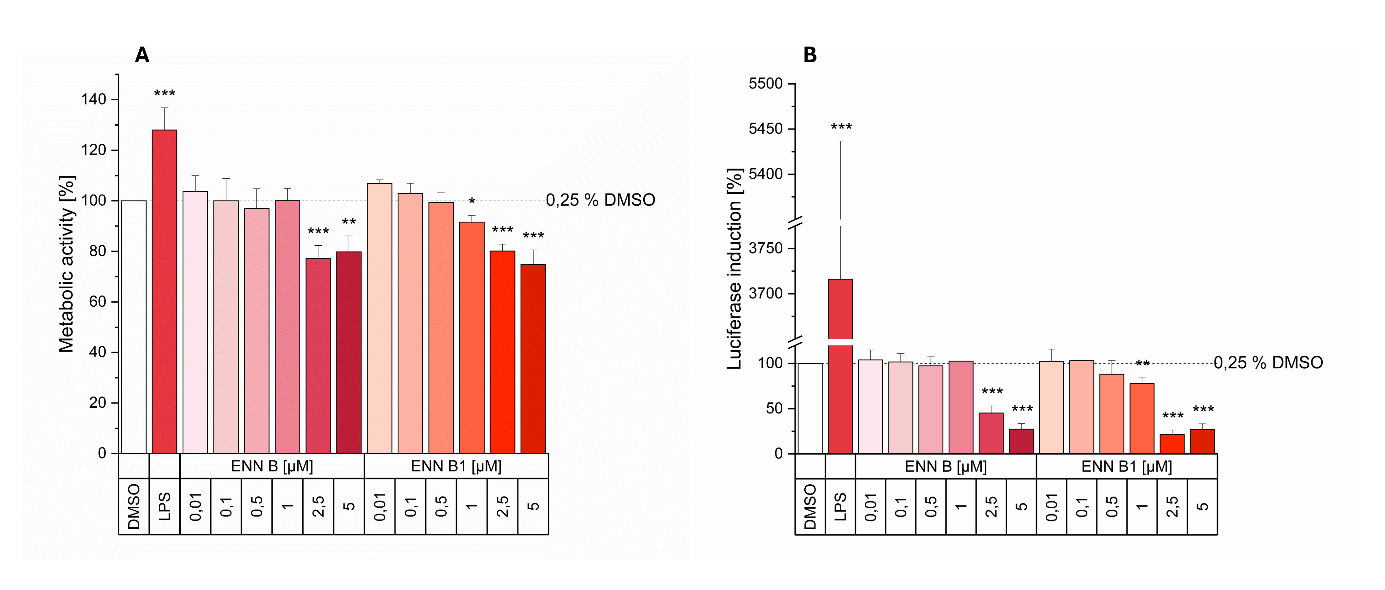


**Figure 2**. Impact of enniatin B and B1 (ENN) **A**) cell viability and **B**) NF-κB signal transduction pathways in non–LPS-stimulated THP1-Lucia™ cells after 20 h incubation. Values were normalized to the control (0.25 % DMSO) as 100 %. Results are depicted as mean + standard deviation of n ≥ 3 biological replicates, each performed in technical triplicates. Significant differences between mycotoxins and the control are indicated by *(p<0.05), **(p < 0.01) and ***(p < 0.001).

1. Characterization of ENN B, ENN B1 and BEA metabolites in S9 rat liver incubations


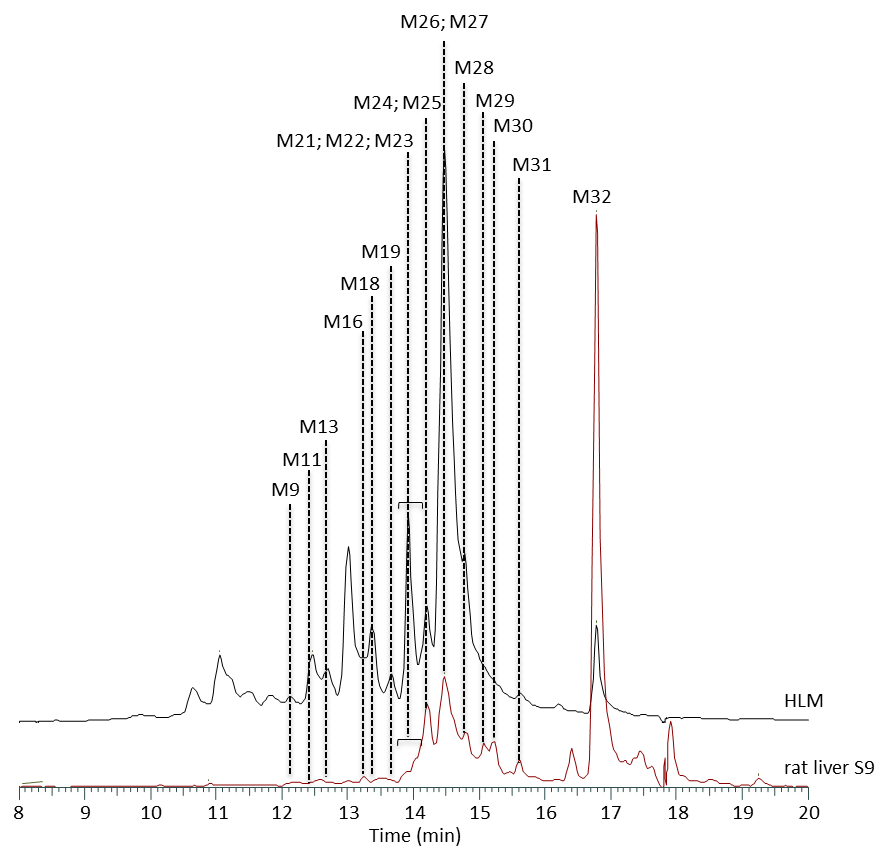


**Figure 3**. Representative extracted ion chromatogram showing ENN B-derived metabolites ([M+Na]^+^) formed in human liver microsomes (HLM) and rat liver S9.

**
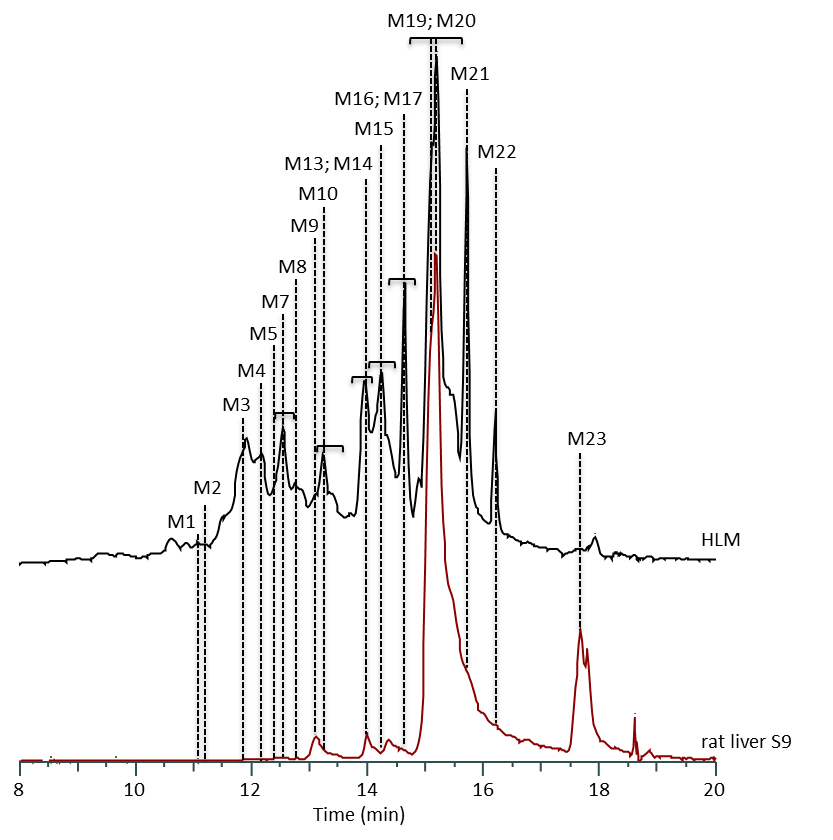
**

**Figure 4**. Representative extracted ion chromatogram showing ENN B1-derived metabolites ([M+Na]^+^) formed in human liver microsomes (HLM) and rat liver S9.

**
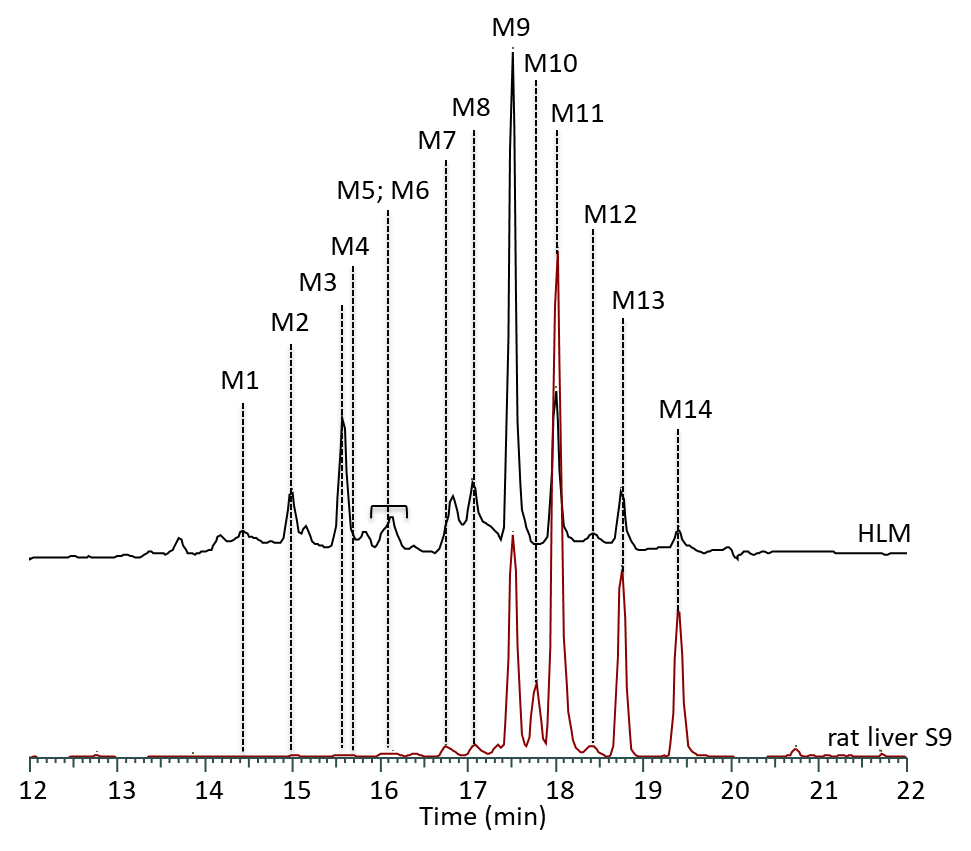
**

**Figure 5**. Representative extracted ion chromatogram showing BEA-derived metabolites ([M+Na]^+^) formed in human liver microsomes (HLM) and rat liver S9.
